# The Surge of Hypervirulent ST398 MRSA Lineage With Higher Biofilm-Forming Ability Is a Critical Threat to Clinics

**DOI:** 10.3389/fmicb.2021.636788

**Published:** 2021-03-04

**Authors:** Huiying Lu, Lin Zhao, Yuanguo Si, Ying Jian, Yanan Wang, Tianming Li, Yingxin Dai, Qian Huang, Xiaowei Ma, Lei He, Min Li

**Affiliations:** ^1^Department of Laboratory Medicine, School of Medicine, Renji Hospital, Shanghai Jiao Tong University, Shanghai, China; ^2^Department of Laboratory Medicine, Qingdao Hiser Medical Center, Qingdao, China

**Keywords:** methicillin-resistant *staphylococcus aureus*, sequence type 398, phylogenetic analysis, whole-genome sequencing, virulence

## Abstract

The global increase of community-associated (CA) infections with methicillin-resistant *Staphylococcus aureus* (MRSA) is a major healthcare problem. Although sequence type (ST) 398 MRSA was first described as a livestock-associated (LA) lineage, human-adapted MRSA (HO-MRSA) ST398 without livestock contact has subsequently been reported from China in our previous study and other later research. The proportion of ST398 HO-MRSA has also remarkably increased in recent years in China. Based on 3878 *S. aureus* isolates that were collected in a general hospital between 2008 and 2018, we identified 56 ST398 HO-MRSA isolates. The four early appearing isolates of them have been sequenced by whole-genome sequencing (WGS) in our previous study. Here, by usage of WGS on the later-appearing 52 isolates and analyzing the phylogenetic dynamics of the linage, we found that 50 isolates clustered together with the former 4 isolates, making it a main clade out of MSSA clones and other MRSA clones, although ST398 HO-MRSA evolved with multiple origins. Drug resistance and virulence gene analysis based on the WGS data demonstrated that ST398 HO-MRSA main clade exhibited a similar pattern in both parts. Furthermore, they all carried a conserved variant of prophage 3 to guarantee virulence and a short SCC*mec* type V element of class D to maintain considerable lower methicillin resistance. Further phenotypical research verified that the epidemic HO-MRSA ST398 displayed enhanced biofilm formation ability when keeping high virulence. The dual advantages of virulence and biofilm formation in the HO-MRSA ST398 subtype promote their fitness in the community and even in the healthcare environment, which poses a serious threat in clinical *S. aureus* infections. Therefore, further surveillance is required to prevent and control the problematic public health impact of HO-MRSA ST398 in the future.

## Introduction

Methicillin-resistant *Staphylococcus aureu*s (MRSA) is not only a human pathogen causing a variety of infections, such as skin and soft tissue infection (SSTI), pneumonia, and sepsis, but it also can colonize and cause diseases in multifarious animals, known as livestock-associated MRSA (LA-MRSA) (Chuang and Huang, 2015; Chen and Huang, 2018). MRSA sequence type (ST) 398 was first identified as a LA-MRSA and also the most dominant clone of LA-MRSA globally, which was first found as prevalent in Europe and then identified outside Europe, including the North America and some Asian countries (Lewis et al., 2008; Larsen et al., 2017).

LA-MRSA has been found more and more in humans and is associated with serious diseases and even death. In the past decade, several infections caused by ST398 MRSA have been reported in the community, ranging from mild skin infections to serious invasive infections and even death, both with and without livestock contact. Human colonization with LA-MRSA ST398, which is genetically identical with LA-MRSA, was first recognized among swine farmers in France and The Netherlands in the early 2000s (Armand-Lefevre et al., 2005; Voss et al., 2005) and showed rapid emergence in Europe (Khanna et al., 2008; Smith et al., 2009, 2013), yet human cases of LA-MRSA ST398 were rarely reported from Asian countries. In contrast, human cases of MRSA ST398 without livestock contact, named host-adapted MRSA ST398 (HO-MRSA ST398), have been gradually reported as emerging in China. Recently, we reported the emergence in Shanghai, China, of several high-virulence HO-MRSA ST398 isolates, which evolved from methicillin-sensitive *Staphylococcus aureus* (MSSA) and are genetically different from LA-MRSA with the characteristics of the lack of *tetM* resistance determinants and the presence of a variant of prophage 3 (He et al., 2018). Soon after, two cases of surgical site infections caused by highly virulent HO-MRSA ST398 were reported in Zhejiang, China (Sun et al., 2019). Subsequently, HO-MRSA ST398 strains has been reported to spread locally in sanatoriums in Taiwan District, probably after entrancing from mainland China (Huang and Chen, 2020).

We continuously tracked the epidemiology of all *S. aureus* isolates in the general teaching hospital and found a rapid increase of ST398 in China while the previously dominant HA-MRSA ST239 clones showed a significant decrease, leading to a marked decrease in the prevalence of MRSA over the past decade (Dai et al., 2019). This prompted us to study those strains to better understand the cause of and the prospect for the rise of HO-MRSA ST398 in China. Here, we report the detection, phylogenetic analysis, epidemiological information, and the phenotypic characteristics of the epidemic HO-MRSA ST398 isolates identified by our clinical laboratory, providing evidence to inform and benefit the clinical prevention and control of *S. aureus* infections.

## Results

### Surveillance of MRSA Isolates at a General Teaching Hospital in 2008–2018

**FIGURE 1:**
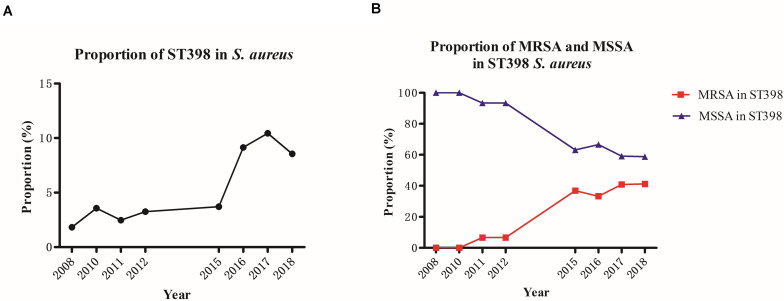
Prevalence of the total ST398 **(A)** and the increasingly emerging dominant MRSA ST398 clone **(B)** from different clinical specimens of patients, 2008–2018.

A total of 2,588 MRSA isolates were identified among 3878 *S. aureus* infections at an affiliated tertiary hospital from 2008 to 2018 in Shanghai, China. The proportion of MRSA infections decreased from 83.5% (643 of 770 total *S. aureus* infections) in 2008 to 49.1% (195 of 397) in 2018. The prevalence of one predominant healthcare-associated MRSA (HA-MRSA) lineage, ST239, significantly decreased (from 48.5 to 4.1%, 2008–2018), which has been proved to be responsible for the marked decrease in the prevalence of MRSA over the past decade. The majority of the 195 MRSA isolates belonged to ST5 (*n* = 99), followed by the community-acquired MRSA (CA-MRSA) clones ST59 (*n* = 26), ST1 (*n* = 21), and ST398 (*n* = 14) in 2018. The proportion of another predominant HA-MRSA lineage, ST5, maintained stability between 2008 (50.1%) and 2018 (50.8%). Contrastingly, the CA-MRSA clones ST59, ST1, and ST398 increased significantly from 0.6% (4 of 643) to 13.3% (26 of 195), 0.5% (3 of 643) to 10.8% (21 of 195), and 0.0% (0 of 643) to 7.18% (14 of 195), respectively, from 2008 to 2018. ST398 MRSA ranked as the third most prevalent CA-MRSA lineage in all SA isolates in 2018. The majority of the total SA isolates belonged to ST5 (*n* = 114) and ST398 (*n* = 34), followed by ST59 (*n* = 32) in 2018. The proportion of ST5 isolates in all SA isolates maintained relative stability between 2008 (41.9%) and 2018 (28.7%), whereas the proportion of ST398 isolates increased obviously from 1.8% (14 of 770) in 2008 to 8.6% (34 of 397) in 2018 (*P* < 0.001), in which it ranked first in prevalence among CA-SA lineage in all SA isolates in 2018 (Figure 1 and Supplementary Table S1). The above data indicate that the increasing emerging dominant MRSA ST398 could be a factor responsible for the remaining stubborn MRSA in recent years.

### Phylogenetic Tree of ST398 MRSA Isolates

**FIGURE 2:**
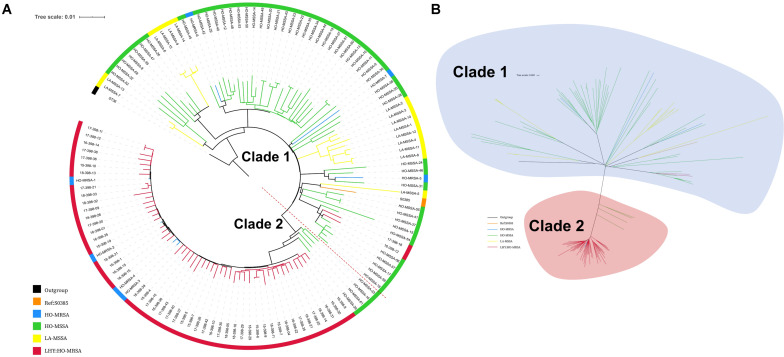
Phylogenetic structure of ST398 *S. aureus* isolates. **(A)** Maximum-likelihood phylogenetic tree of ST398. A total of 129 *S. aureus* ST398 isolates (114 from adult patients and 15 from livestock) were used for phylogenetic reconstruction. Relationships are shown with respect to the genome of the standard LA-MRSA strain (S0385). Branches are colored by geographic origin of isolates. The tree is rooted with ST36 as an outgroup. **(B)** Unrooted tree of ST398 *S. aureus* isolates. Branches in this tree could be classified into two main clades, including Clade I and Clade II. Branches are colored by geographic origin of isolates. Black branch: outgroup. Orange branch: reference isolate. Blue branch: human-adapted (HO-MRSA) isolates sequenced in our previous study. Green branch: HO-MSSA isolates in our previous study. Yellow branch: livestock-adapted (LA-MSSA) isolates in our previous study. Red branch: HO-MRSA isolates sequenced in this study.

A total of 56 MRSA ST398 isolates were collected in this study, while 4 of them that were collected during 2012–2014 had already been analyzed in our previous study (He et al., 2018). Therefore, 52 ST398 MRSA isolates collected during 2015--2018 were whole genome sequenced in this study. The Illumina sequences generated in this study are deposited and available in the Sequence Read Archive (SRA)^1^ under the study accession number PRJNA624723 with the SRA accession numbers SRR11526821 to SRR11526872. We also included our previous genomic data of 61 ST398 isolates (sample accession numbers: SRR5054902 to SRR5054977 and SRR5062006 in the SRA of NCBI). All the characteristic data of 113 ST398 *S. aureus* isolates used for phylogenetic analysis are listed in Supplementary Table S2. The core-genome SNPs were applied for phylogenetic tree reconstruction based on the data of 52 current MRSA ST398 genome sequences and our previously published data of 61 ST398 genome sequences (7 HO-MRSA isolates and 54 HO-MSSA isolates during 2010–2015) using maximum likelihood estimation (Figure 2). All ST398 isolates were divided into two major phylogenetic clades, with each supported by 100% bootstrapping. Most of the HO-MSSA ST398 strains were in Clade 1. However, there were also five human-adapted MRSA ST398 strains in Clade 1, including 17-398-18, 16-398-12, HO-MRSA-5, HO-MRSA-6, and HO-MRSA-7. Human-adapted MRSA ST398 were mainly in Clade 2, including 4 HO-MRSA ST398 isolates from our previous study, clustered with the four isolates of HO-MSSA ST398 isolates. Clade 1 was a mixture of samples collected from both humans and livestock, while Clade 2 was a pure clade of sample from humans, which accounted for 91.7% (55/60) of HO-MRSA ST398 isolates sampled, suggesting that the epidemic MRSA ST398 mainly originated from Clade 2. The above data showed that the increasing emerging dominant MRSA ST398 in Shanghai, China, mainly evolved to a subclone.

### Clinical Characteristics of ST398 HO-MRSA

Among the 56 MRSA ST398 isolates collected in this study, 96.42% (54 of 56) were identified as HO-MRSA according to the phylogenetic tree. Only two isolates (17-398-18 and 16-398-12) were finally judged as LA-MRSA originated, which clustered with S0385, and clinical data also showed that these two patients ever had livestock contact. Interestingly, 3 of 56 infection cases (18-398-11, 18-398-21, and 18-398-32) by ST398 MRSA that clustered in Clade 2 were found to occur > 48 h after hospitalization in 2018, which suggested the onset of high-virulence MRSA ST398 in the healthcare environment.

### Antibiotic Susceptibility

We profiled the 113 ST398 isolates with *in vitro* susceptibility tests to 13 common antibiotics. Almost all the ST398 *S. aureus* isolates were susceptible to RD, TEC, LZD, and VA and resistant to P (antibiotics abbreviations provided in the “Materials and Methods” section). Compared to the 54 ST398 MSSA, the 56 ST398 MRSA collected in this study together with 3 ST398 MRSA from other hospitals displayed total resistance to FOX and high resistance rates for CZ (41 of 59; 69.49%) (Supplementary Table S2), whereas ST398 MSSA exhibited higher resistance rates for CN (14 of 54, 25.93%) and FOS (6 of 54, 11.11%), compared with ST398 MRSA (1 of 59, 1.69% and 0 of 59, 0.00%).

### Virulence Factors and Resistance Gene Analysis

**FIGURE 3:**
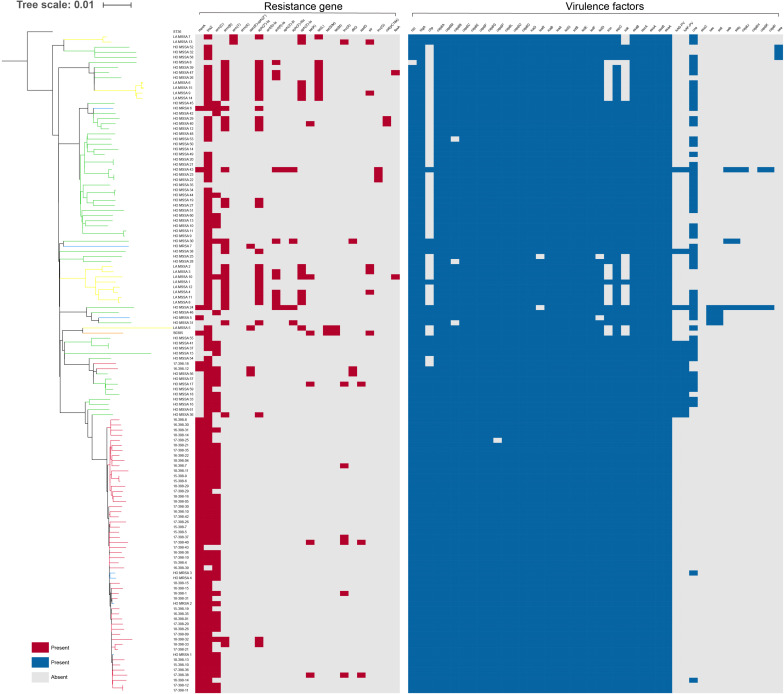
Presence or absence of antibiotic resistance genes and virulence genes among ST398 isolates. The distribution of antibiotic resistance genes and virulence genes is plotted against the core genome phylogeny; the presence of an antibiotic resistance or virulence gene in an isolate is shown in red and blue, respectively, and the absence is shown in gray.

We further mapped virulence and antibiotic susceptibility information for each isolate to the phylogeny (Figure 3). In all ST398 strains, 20 virulence genes were found, including *hlgA*, *hlgB*, *hlgC*, *scn, sak, hla*, *aur, icaA*, *icaB*, *icaC*, *icaD*, *clfA*, etc. Among the 20 virulence genes, *hlgA*, *hlgB*, and *hlgC* encode γ-hemolysin; staphylococcal complement inhibitor (*scn*) is associated with immune evasion; *sak* encodes staphylokinase, contributing to spread of the bacteria; and *aur* encodes aureolysin, which belongs to staphylococcal exoenzyme. All the HO-MRSA ST398 strains in Clade 2 harbored the *scn*, staphylokinase (*sak*), and chemotaxis inhibitory protein (*chp*) genes, the three of which are in common with the conserved variant of prophage 3. Almost none of the above strains carried the *pvl* gene or enterotoxin genes or *cna* gene. Nevertheless, other HO-MRSA ST398 strains scattered in Clade 1 carried only *scn* and *sak* genes, and the *chp* gene was always absent in this clade.

Antibiotic resistance genes were rarely found in all ST398 strains. All the HO-MRSA ST398 isolates in Clade 2 carried *mecA* (methicillin resistance gene). Almost all of them harbored *blaZ* (penicillin resistance gene) and *erm*(C) (macrolide resistance gene). Besides the above genes, the HO-MRSA ST398 isolates in Clade 2 carried very few other antibiotic resistance genes. The strains in Clade 1 also harbored very few antibiotic resistance genes besides *blaZ*.

The above data suggested that all the HO-MRSA ST398 strains in Clade 2 had similar antibiotic resistance and virulence pattern background with the conserved variant of prophage 3, containing the immune evasion complex (IEC) genes encoding the chemotaxis inhibitory protein (CHIP), staphylococcal complement inhibitor (SCIN), and staphylokinase (SAK) together, which was usually different from that of HO-MRSA ST398 in Clade 1, thus probably facilitating the clone transmission of this specific ST398 MRSA.

### SCC*mec* Analysis

**FIGURE 4:**
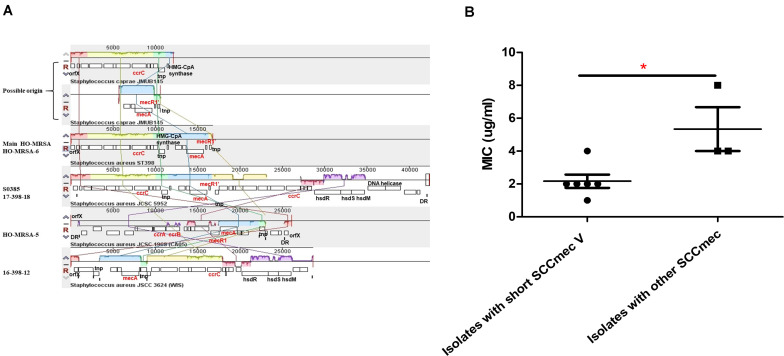
Analysis of HO-MRSA ST398 SCC*mec*. **(A)** SCC*mec* comparison. **(B)** Comparison of oxacillin MIC. *p* ≤ 0.05 (*).

The most interesting feature of the genome of all HO-MRSA ST398 isolates in Clade 2 and one HO-MRSA ST398 isolate in Clade 1 (HO-MRSA-6) is the SCC*mec* type V element of class D in *S. aureus* only described in our previous study (He et al., 2018), characterized by an IS431-*mecA*-*mecR1*’ composition and one copy of the *ccrC* recombinase gene, which has previously only been found in CoNS like *S. caprae* (Katayama et al., 2001). However, other HO-MRSA ST398 isolates in Clade 1 have different SCC*mec* elements, respectively. One HO-MRSA ST398 strain (17-398-18) harbors SCC*mec* type V (5C2&5) (composition IS431-*mecA*-*mecR1’*-IS431 and two *ccrC* copies), which belong to most LA-MRSA ST398 strains, including the reference strain S0385. Another HO-MRSA ST398 strain (HO-MRSA-5) contains SCC*mec* type IVa (2B) with the following gene complexes: *ccrA2-ccrB2* and *mecA-mecR1*. Furthermore, the HO-MRSA ST398 strain (16-398-12) harbors SCC*mec* type V (5C2) with the following gene complexes: *ccrC* and *mecA* (Figure 4A). Obviously, the SCC*mec* type V element of class D is shorter than other kinds of SCC*mec* elements in Clade 1.

Considering previous findings (Pozzi et al., 2012; Rudkin et al., 2012) that have shown repression of virulence by the methicillin resistance-encoding *mecA* gene and our previous results (He et al., 2018) showing that striking and highly conserved differences of MICs to β-lactams and *mecA* expression exist between HA-MRSA clones and CA-MRSA clones, here we further sought for the possible influence of this kind of SCC*mec* to MIC of oxacillin and *mecA* expression. Six HO-MRSA ST398 isolates with short SCC*mec* V were randomly selected to compare the MIC of oxacillin with the isolates with other SCC*mec*. Isolates with the short SCC*mec* V showed lower oxacillin MIC levels compared to other CA-MRSA-characteristic SCC*mec* types (Figure 4B) in accord with our previous finding (He et al., 2018), showing that this SCC*mec* element in epidemic HO-MRSA ST398 confers only very low-level methicillin resistance to fit both in the community and healthcare environment where the former epidemic HA-MRSA ST239 with high methicillin resistance is significantly decreasing now.

The above data indicated that the short SCC*mec* V element of class D could be a factor accounting for the fitness of HO-MRSA ST398 strains in Clade 2 both in the community and healthcare setting.

### HO-MRSA ST398 Epidemic Isolates Exhibit High Virulence

**FIGURE 5:**
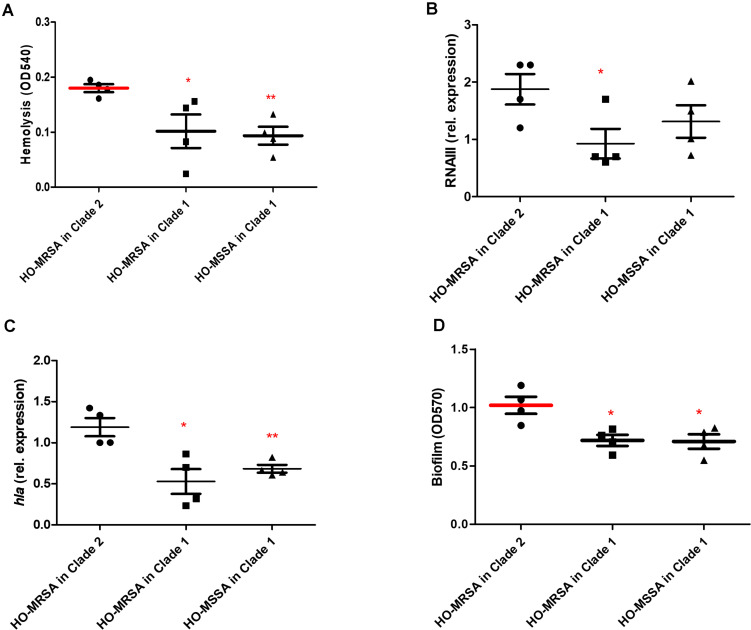
Phenotypic differences between HO-MRSA ST398 in Clade 1 and Clade 2. **(A)** Hemolysis (erythrocyte lysis) of four representative HO-MRSA ST398 isolates, respectively, in Clade 1 and Clade 2. Hemolytic activities were determined by incubating culture filtrates with human red blood cells. **(B,C)** Expression of *RNAIII*/*hla* was determined by qRT-PCR. **(D)** Biofilm elaboration measured using polystyrene microtiter plate assay. Statistical analysis was performed using Student’s *t*-test. The data shown are mean ± SD of three independent experiments. Statistical significances: *p* > 0.05 (ns), *p* ≤ 0.05 (*), *p* < 0.001 (**).

We randomly selected four representative HO-MSSA ST398 isolates from Clade 1 and four representative HO-MRSA ST398 isolates from Clade 2, together with four HO-MRSA ST398 isolates in Clade 1. We next measured cytolytic potential by lysing analysis of human erythrocytes and determined expression of RNAIII and α-toxin as important core genome-encoded virulence determinants (Figures 5A–C). HO-MRSA ST398 in Clade 2 displayed high erythrocyte lysis capacity and also exhibited elevated RNAIII and α-toxin expression at the transcriptional level in comparison with the other two groups in Clade 1. All these analyses showed that the HO-MRSA ST398 isolates in Clade 2 have higher virulence compared to the HO-MRSA ST398 isolates and the closely related MSSA isolates in Clade 1.

### HO-MRSA ST398 Epidemic Isolates Exhibit Higher Biofilm Formation Ability

In addition to the observed high virulence, semi-quantitative biofilm tests of the above 12 representative ST398 isolates revealed that compared with the 4 HO-MRSA and 4 HO-MSSA ST398 isolates in Clade 1, the 4 HO-MRSA ST398 isolates in Clade 2 demonstrated significantly higher biofilm formation, the key process in the occurrence and development of persistent infections (Figure 5D).

## Discussion

To our knowledge, this is the first report focusing on the epidemiologically important emerging host-adapted MRSA ST398 in China. Based on our previous study and this study, the first ST398 isolate was identified in 2011 from a 65 year-old male patient whose sputum and blood cultures grew MRSA ST398, which suggests that ST398 existed in Shanghai since 2011 or earlier. Also, the population of both ST398 and MRSA ST398 gradually increased annually in Shanghai, China.

MRSA ST398, first identified as a LA-MRSA, was prevalent in Europe and the North America. However, it was rarely reported in Asian countries (Lewis et al., 2008; Chuang and Huang, 2015; Larsen et al., 2017; Chen and Huang, 2018). Human-adapted ST398 isolates, which are different from LA clones in genetics, often cause life-threatening infections and have some common genetic characteristics in adapting to human as host (Price et al., 2012; Larsen et al., 2016; He et al., 2018; Sun et al., 2019). Most previous reports found that human-adapted ST398 isolates are MSSA (Zhao et al., 2012). In our recent report (He et al., 2018), eight cases of ST398 CA-MRSA infection were reported in China, of which six cases were severe and two cases died. Genome analysis showed that these highly infectious strains of ST398 CA-MRSA evolved from human-adapted methicillin-sensitive clones, which were then called ST398 HO-MRSA. Similar severe and even fatal cases have also been reported in Japan and Australia (Koyama et al., 2015; Coombs et al., 2019). Subsequently, 12 strains of MRSA ST398 were identified as colonization strains in Taiwan (Huang and Chen, 2020). The strain appears to be spreading internationally and needs further monitoring.

Phylogenetic reconstructions of the dominating pandemics of healthcare-associated ST239 MRSA and LA ST398 MRSA have been well described (Harris et al., 2010; Price et al., 2012; Hsu et al., 2015), while the evolutionary dynamics of the increasing ST398 HO-MRSA has rarely been studied. We identified 52 ST398 MRSA during 2015 and 2018 in the general hospital in Shanghai, China, together with six ST398 MRSA during 2011–2014 in our previous study. Next, the phylogeny of ST398 MRSA linages using the above 58 individual ST398 MRSA isolates was reconstructed, proving that ST398 MRSA isolates evolved with multiple origins and 54 of them evolved together as an emerging main clade that differed from those MSSA isolates in clinical *S. aureus* infections. ST398 MRSA isolates in the main Clade (Clade 2) definitely exhibited differences both genetically and phenotypically in comparison to ST398 MSSA and other ST398 MRSA isolates clustered in Clade 1.

Herein, analysis of the virulence genes and the resistance genes revealed that ST398 MRSA isolates in Clade 2 exhibited uniform virulence and antibiotic resistance pattern. Discrepancy in the virulence genes between ST398 MRSA in Clade 2 and ST398 MRSA and ST398 MSSA in Clade 1 mainly focused on the *chp*, which is conserved in Clade 2, while all of them harbor the *scn* and *sak genes*. The CHIP encoded by *chp* is a 14 kDa protein that blocks neutrophil chemotaxis via binding the formylated peptide receptor and the C5a receptor on neutrophils (Postma et al., 2004, 2005). Higher carriage rate of *chp* in these isolates might indicate a higher ability of immune escape of ST398 HO-MRSA main clade during host defense in *S. aureus* infections. In addition to the high presence of virulence genes, ST398 HO-MRSA isolates displayed higher hemolysis capacity, making the ST398 HO-MRSA in Clade 2 a hypervirulent linage in clinical *S. aureus* infections. On the other hand, ST398 HO-MRSA isolates exhibited high sensitivity to several common antibiotics as well as ST398 HO-MSSA isolates. Additionally, the type of all the ST398 HO-MRSA in Clade 2 is the short SCC*mec* type V element of class D, which maintained the much lower methicillin resistance.

Generally speaking, hypervirulent isolates exhibited higher susceptibility to the most common antibiotics, while multi-drug resistant (MDR) isolates would be less virulent, which is named “fitness cost” in related research (Foucault et al., 2009; Maher et al., 2012; Nielsen et al., 2012). However, strains with high virulence have appeared under scrutiny recently in the healthcare environment (Rasko and Sperandio, 2010). Herein, this study shows that ST398 HO-MRSA in Clade 2 displayed lower resistance to methicillin and several common antibiotics while exhibiting higher virulence in comparison to ST398 HO-MSSA and ST398 HO-MRSA in Clade 1. Moreover, ST398 HO-MRSA isolates in Clade 2 formed thicker biofilm compared to the isolates in Clade 1, which was more common in healthcare-associated *S. aureus* infections, promoting the fitness of the ST398 HO-MRSA in the community and even hospital. Thus, more attention should be paid to the ST398 HO-MRSA isolates in the prevention and control of infections.

As for the limitations of this study, since all the ST398 *S. aureus* isolates identified and characterized in this study were only from an affiliated tertiary hospital in Shanghai, further studies are needed to determine the whole perspective of HO-MRSA ST398 *S. aureus* in China.

In conclusion, our data provide important insight into the current epidemic status, pathogenicity, transmission, and phylogenetic relationship of the human-adapted MRSA ST398 in Shanghai, China. Genomic analyses presented here, in conjunction with the epidemiological data, suggest that the epidemic transmission of HO-MRSA ST398 is strongly related to the short SCC*mec* type V element maintaining the much lower methicillin resistance and the conserved variant of prophage 3, as well as containing the complete IEC genes, guaranteeing virulence. Further phenotypical research verified that the epidemic HO-MRSA ST398 showed higher biofilm formation ability when maintaining high virulence, promoting their fitness in the community and even in the healthcare environment. Additional research and surveillance are required to predict the public health impact of HO-MRSA ST398 in the future.

## Materials and Methods

### Bacterial Strains, Growth Conditions, and Clinical Definitions

*S. aureus* strains were grown in tryptic soy broth (TSB) (Oxoid) with 0.25% glucose or on tryptic soy agar plates at 37°C. We collected and analyzed a total of 3,878 clinical isolates from adult patients at a comprehensive teaching hospital in Shanghai, China, between 2008 and 2018, from which the epidemiological data of 3695 clinical isolates between 2008 and 2017 have been published in our previous study (Dai et al., 2019).

ST398 MRSA and MSSA isolates were further investigated in the present study after initial characterization. Healthcare-associated SA (HA-SA) was defined as SA infection that occurred > 48 h after hospitalization. Community-associated SA (CA-SA) was defined as an isolate that was obtained either from an outpatient or from an inpatient (including those from general and urgent care and emergency rooms) ≤ 48 h after hospital admission. Human-adapted SA (HO-SA) was defined as human-originated and adapted SA without livestock contact, which is genetically different from LA-SA (He et al., 2018).

An infection was considered invasive when isolates were isolated from otherwise sterile body sites. Clinical data were obtained from patient electronic medical records. The information collected included the location of the patient at the time of sample collection, date of sample collection, date of isolation, and body site of the sample. The clinical syndrome of MRSA infection was classified into syndrome categories (SSTI, respiratory infection, bacteremia, CSF, or other sterile body fluid).

### Antimicrobial Resistance Profiles

Antibiograms were determined by standard disc diffusion on Mueller–Hinton agar in accordance with the Clinical and Laboratory Standards Institute (CLSI) guidelines. Fourteen antimicrobial agents tested included gentamycin (CN), penicillin (P), cefazolin (CZ), erythromycin (E), clindamycin (DA), sulfamethoxazole-trimethoprim (SXT), fosfomycin (FOS), rifampicin (RD), levofloxacin (LEV), cefuroxime (CXM), teicoplanin (TEC), linezolid (LZD), vancomycin (VA), and cefoxitin (FOX). *S. aureus* ATCC29213 was used as a quality control.

### Molecular Typing

Molecular typing was performed using multilocus sequence typing (MLST) as previously described (Maiden et al., 1998). The sequences of the polymerase chain reaction (PCR) products were compared with the existing sequences available at the MLST website^2^.

### Whole-Genome Sequencing of ST398 Isolates and Genome Comparison

Chromosomal DNA of 52 *S. aureus* ST398 isolates was extracted by a standard phenol–chloroform extraction procedure. *S. aureus* whole-genome sequencing was performed on an Illumina HiSeq 2500 sequencer (Illumina, San Diego, CA, United States) with 150 bp paired-end reads. The data generated from the Illumina platform were analyzed after quality control was performed.

Original sequencing reads were exported to Fastq files, and then snippy (BWA, SAMtools, SnpEff, and Freebayes) was used to align reads to the S0385 ST398 chromosome as a reference [GenBank:AM990992] to generate the SNPs of the core genome. ST36 [GenBank:BX571856], determined by Price et al. as the most closely related non-CC398 ST (Price et al., 2012), was used as an outgroup. The repeat regions were removed by TRF and BLASTN. Gubbins was used to remove the recombinant region in the genome, and the total number of SNPs was 28,644, and then SNPs only caused by outgroup were eliminated.

Fastq files of 76 ST398 samples including 7 HO-MRSA, 54 HO-MSSA, and 15 LA-MSSA ST398 isolates and in our previous study (He et al., 2018) were also downloaded from GenBank, and the variants were also called using the preceding strategy. Therefore, the total number of SNPs was 7205.

### Phylogenetic Analysis

The maximum likelihood tree was constructed based on the 7205 SNPs in the core genome after duplicate and recombination reads were removed, using the GTR + G model in the RAxML software by 100 bootstraps. The phylogenetic results were displayed by ITOL^3^ (Letunic and Bork, 2019).

### Genome Assembly and Detecting the Presence of Virulence-Associated Genes and Antibiotic Resistant Genes

Pre-processed reads were *de novo* assembled using CLC Genomics Workbench 12.0 (Qiagen) using the default options. Then, the generated *de novo* assembled contigs were analyzed separately via the pipelines of BLASTing the drug resistant gene database (Resfinde database, 2020-05-28) (Zankari et al., 2012; Liu et al., 2019) and at the Virulence FactorsDatabase (Larsen et al., 2012; Liu et al., 2019) in the CLC software.

### SCC*mec* Identification

The SCC*mec* element is the defining feature of MRSA isolates and encodes the single determinant for methicillin resistance, the *mecA* gene. In order to assign SCC*mec* type, we use SCC*mec*Finder, which identifies SCC*mec* elements in sequenced *S. aureus* isolates^4^.

### Lysis of Erythrocytes by Culture Filtrates

Supernatants were collected from bacterial cultures grown for 15 h. Hemolytic activities were determined by incubating samples with human red blood cells (2% v/v in Dulbecco’s phosphate-buffered saline, DPBS) for 1 h at 37°C. Hemolysis was determined by measuring the optical density at 540 nm using an enzyme-linked immunosorbent assay (ELISA) reader. The assay was performed in triplicate.

### Quantitative Reverse-Transcription (qRT)-PCR

Overnight cultures were diluted 1:100 into 50 ml of TSB and incubated at 37°C with shaking at 200 rpm until grown to mid-exponential phase (4 h). Complementary DNA was synthesized from total RNA using the QuantiTect Reverse Transcription Kit (Qiagen) according to the manufacturer’s instructions. Oligonucleotide primers were designed using Primer Express. The primers used are listed in Supplementary Table S1.

The resulting complementary DNA and negative control samples were amplified using the QuantiTect SYBR Green PCR Kit (Qiagen). Reactions were performed in a MicroAmp Optical 96-well reaction plate using a 7500 Sequence Detector (Applied Biosystems). Relative messenger RNA (mRNA) levels were calculated using *gyrB* as a control. All qRT-PCR experiments were performed in duplicate.

### Semi-Quantification of Biofilms

Crystal violet staining was applied to semi-quantify biofilm formation of *S. aureus* strains. Briefly, overnight bacterial cultures were diluted into TSBg to a final optical density of 0.05. The diluted cultures were aliquoted to 96-well flat-bottom tissue culture plates (200 μl/well) and incubated at 37°C for 24 h. Wells were washed with PBS after gentile removal of culture supernatants. Bouin fixative was added onto the bottom of the wells to treat biofilm for 1 h. The fixative was gently aspirated out and wells were washed three times with PBS and then stained with 0.4% (wt/vol) crystal violet. Biofilm formation was measured by a MicroELISA autoreader (BioTeK, United States) at 570 nm.

### Growth Curve

Growth curves were performed as previously described. Overnight cultures were diluted by 100-fold into fresh TSB media and incubated at 37°C under shaking conditions for 24 h. OD_600_ was measured every 2 h.

### Statistics

Statistical analysis was performed using GraphPad Prism v6.0. For the comparison, unpaired, two-tailed *t*-tests were used. All error bars depict the standard deviation. Lines depict the mean.

## Data Availability Statement

The Illumina sequences generated in this study are deposited and available in the Sequence Read Archive (SRA) (http://www.ncbi.nlm.nih.gov/sra) under the study accession number PRJNA624723 with the SRA accession numbers SRR11526821 to SRR11526872.

## Ethics Statement

Written informed consent was obtained from the individual(s) for the publication of any potentially identifiable images or data included in this article.

## Author Contributions

ML and LH contributed to the conception and design of the study, and wrote the first draft of the manuscript. HL, LH, YS, and XM performed all the experiments. HL and LZ analyzed the statistics and plotted the figures and tables in this work. YJ, YW, TL, QH, and YD wrote sections of the manuscript. All authors contributed to manuscript revision and read and approved the submitted version.

## Conflict of Interest

The authors declare that the research was conducted in the absence of any commercial or financial relationships that could be construed as a potential conflict of interest.

## Footnotes

1 http://www.ncbi.nlm.nih.gov/sra

2 https://pubmlst.org/multilocus-sequence-typing

3 https://itol.embl.de/

4 https://cge.cbs.dtu.dk/services/SCCmecFinder/
